# Functional and Structural Characterization of the *Erythrina fusca* Seed Lectin EFusL: A GalNAc-Binding Lectin with Specificity for Tumor-Associated O‑Glycans

**DOI:** 10.1021/acsomega.6c05946

**Published:** 2026-08-18

**Authors:** Messias V. Oliveira, Francisco W. V. Martins, Clara Suarez, Valeria M. S. Ferreira, Vanir R. Pinto-Junior, Vinicius J. S. Osterne, Luiz A. G. Souza, Els J. M. Van Damme, Rodrigo B. Leal, Benildo S. Cavada, Kyria S. Nascimento

**Affiliations:** † Federal University of Ceara, Department of Biochemistry and Molecular Biology, Pici Campus Prof. Prisco Bezerra, S/N, Pici, Fortaleza 60.455-970, Brazil; ‡ National Institute for Research in the Amazon, Department of Agronomic Sciences, 2936 Andre Araujo Avenue, Petropolis, Manaus 69067-375, Brazil; § Ghent University, Laboratory of Biochemistry and Glycobiology, Department of Biotechnology, Proeftuinstraat 86, Ghent 9000, Belgium; ∥ Federal University of Santa Catarina, Department of Biochemistry, Biological Sciences Center, Block G, 2nd Floor, Room 219, Florianopolis 88037-000, Brazil

## Abstract

Lectins from the genus *Erythrina* are predominantly galactoside-binding legume lectins with conserved structural features and established use as glycan-recognition tools. However, several species remain incompletely characterized, including the seed lectin from *Erythrina fusca* (EFusL). Here, EFusL was isolated by single-step lactose-agarose affinity chromatography and characterized by hemagglutination assays, carbohydrate inhibition, physicochemical stability tests, electrophoresis, mass spectrometry-based sequencing, structural modeling, molecular docking, and toxicity assays. EFusL migrated as a 29–31 kDa glycoprotein and contained approximately 4.3% neutral carbohydrates. Hemagglutination inhibition confirmed a galactoside-directed profile, with lactose, galactose, and *N*-acetyl-d-galactosamine among the effective inhibitors. EFusL showed maximal hemagglutinating activity at pH 8.0, retained activity up to 40 °C, and displayed reduced activity after EDTA treatment with partial recovery following Ca^2+^/Mn^2+^ supplementation. Sequence analysis identified a 249-residue mature lectin highly similar to *Erythrina corallodendron* and *Erythrina cristagalli* lectins, including conservation of residues associated with galactoside recognition. Structural modeling supported the canonical β-sandwich legume lectin fold, and docking analyses indicated that EFusL accommodates galactoside ligands through a conserved polar interaction network comparable to related *Erythrina* lectins and legume lectins in general. Under the tested conditions, EFusL did not induce lethality in *Artemia salina* and did not significantly affect NHDF viability, although moderate reductions in HeLa and HT-1080 viability were observed under selected concentration- and time-dependent conditions. Together, these results define EFusL as a structurally conserved, galactoside-binding *Erythrina* lectin with moderate physicochemical stability and limited cell-type-dependent effects in the evaluated models, supporting its classification within the canonical *Erythrina* lectin group with similar biotechnological potential.

## Graphical abstract

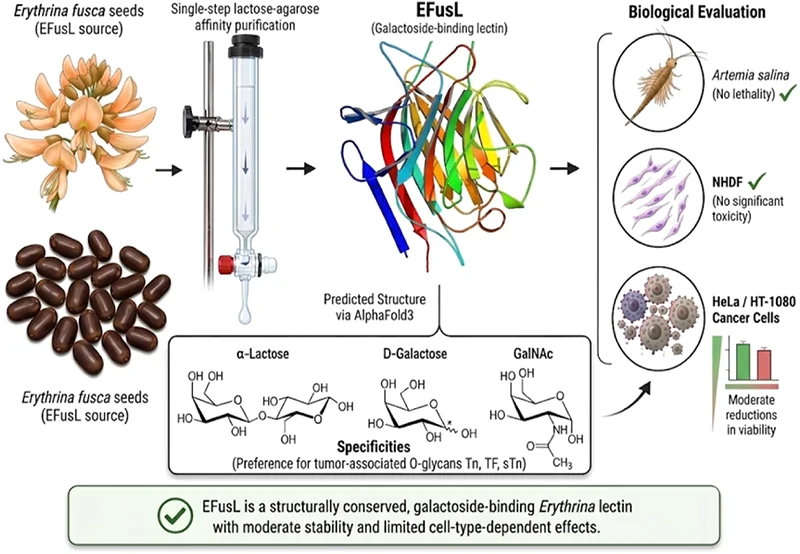

## Introduction

A recurring theme in lectin biology is the tension between conservation and variability: a conserved fold can sustain a shared recognition mode, while subtle sequence differences reshape ligand accommodation and downstream biological interactions.^1,2^ Lectins from the genus *Erythrina* embody this principle, combining a conserved carbohydrate-binding framework with detectable differences in sugar inhibition profiles and cell-related behaviors among species and isoforms.^3^ Such features motivate the characterization of less comprehensively described members of the genus, including the seed lectin from *E. fusca*.

Within the legume lectin family, lectins from the genus *Erythrina* form a well-known group of predominantly galactoside-binding proteins, frequently showing preference for LacNAc-related motifs and being widely used as affinity reagents and histochemical probes for terminal galactosyl determinants.^4^ Beyond their analytical use, *Erythrina* lectins have been associated with diverse biological activities in mammalian systems, most notably hemagglutination and mitogenic stimulation of human peripheral blood lymphocytes, with *Erythrina cristagalli* lectin (ECL) being among the best-characterized examples.^5,6^ In addition, the genus includes lectins applied as markers of differentiation-associated changes in cell-surface glycans and as tools in glycoprotein/glycoform discrimination (e.g., affinity-based fractionation of α-fetoprotein glycoforms relevant to hepatocellular carcinoma),^7^ reinforcing their value for probing biologically meaningful glycosylation patterns. ECL has also been explored in cell-based platforms where lectin–glycan recognition is leveraged as a functional interface, including matrices that support the growth and maintenance of pluripotent stem cells, illustrating how canonical Gal/Lac/LacNAc recognition can be translated into practical biological applications.^8^

Structural studies of *Erythrina* lectins have provided detailed insights into protein–carbohydrate recognition: the lactose complex of the *Erythrina corallodendron* lectin (EcorL) revealed a dimeric lectin architecture and a conserved carbohydrate-binding site that reveals its binding mode.^9^ Importantly, although *Erythrina* lectins share a conserved β-sandwich framework and a highly conserved CRD, homologues can differ in extended-site interactions that modulate fine specificity (e.g., toward fucosylated or LacNAc-related determinants).^10^ Therefore, comparisons among *Erythrina* lectins should distinguish between conservation of the core binding architecture and ligand-dependent differences in fine specificity. Consistent with this view, comparative studies across *Erythrina* species highlight strong sequence conservation and broadly similar recognition profiles, while still allowing measurable differences in inhibition potency and ligand accommodation.^11^

*Erythrina fusca* Lour. is a widely distributed tropical species and a promising source of a biologically active legume lectin.^12^ In Brazil, it grows in swampy and flood-prone areas. It has a thorny bark, light orange flowers, and 20 cm pods with dark brown seeds. Like other *Erythrina* species, it contains toxic alkaloids with medicinal potential.^13,14^ The integrated characterization covering purification, carbohydrate-binding properties, physicochemical stability, sequence analysis, and structural modeling of the lectin from *E. fusca* could be valuable for the legume lectin field.

In this context, we report the purification of an *E. fusca* seed lectin (hereafter EFusL), followed by a systematic assessment of its hemagglutination activity and carbohydrate specificity, stability under pH and temperature variations, and the influence of divalent cations on its activity. In addition, we solve its complete primary structure using mass spectrometry combined with molecular biology approaches and present a high-confidence three-dimensional model predicted via AlphaFold3. Thus, this enables a direct comparison with well-characterized *Erythrina* lectins. Finally, we investigate its effects on human cell lines of biomedical interest in an attempt to expand the functional dataset available for lectins from this genus and galactoside-binding legume lectins in general.

## Materials and Methods

### Chemicals and Reagents

Acrylamide, *N*′,*N*′-methylenebis­(acrylamide), Coomassie Brilliant Blue, TEMED, bovine serum albumin (BSA), EDTA, and carbohydrates used in inhibition assays were purchased from Sigma-Aldrich. SDS and 2-mercaptoethanol were obtained from Merck. Agarose–lactose resin for affinity chromatography and DEAE-Sephacel resin were purchased from GE Healthcare. For cell-based assays, DMEM, fetal bovine serum (FBS), penicillin/streptomycin, and PrestoBlue HS were obtained from Invitrogen (Thermo Fisher Scientific). Fluorescence measurements were performed using an Infinite F200 Pro microplate reader (Tecan). All solutions were prepared using ultrapure water and filtered when required.

### Purification of EFusL

Mature *E. fusca* seeds were ground to a fine powder and extracted at a 1:10 (w/v) ratio (1 g flour per 10 mL) in 20 mM Tris–HCl buffer (pH 8.0) containing 150 mM NaCl. The suspension was stirred for 4 h at room temperature and then centrifuged at 9,000 × *g* for 20 min at 4 °C. The clarified supernatant was filtered through gauze/nylon mesh and designated as crude extract.

The crude extract was applied to an agarose–lactose affinity column (2.0 × 5.0 cm) previously equilibrated with 20 mM Tris–HCl (pH 8.0) containing 150 mM NaCl. The column was washed with equilibration buffer until the absorbance at 280 nm returned to baseline. Bound proteins were eluted with equilibration buffer supplemented with 100 mM lactose. Fractions of 1.5 mL were collected and monitored at 280 nm, and hemagglutinating activity was determined in each fraction. Fractions exhibiting hemagglutinating activity were pooled, dialyzed against deionized distilled water, and freeze-dried.

### Hemagglutination and Sugar-Inhibition Assays

Hemagglutinating activity was assessed in 96-well U-bottom microtiter plates using serial 2-fold dilutions of lectin prepared in 100 mM Tris–HCl buffer (pH 7.6) containing 150 mM NaCl. A 2% (v/v) erythrocyte suspension from rabbit and human erythrocytes was used in the assays. Human erythrocytes from blood groups A, B, and O were obtained from blood bags provided by the Centro de Hematologia e Hemoterapia do Ceará (HEMOCE, Fortaleza, Ceará, Brazil), upon institutional request from BioMol-Lab/UFC. Rabbit erythrocytes were purchased from a commercial supplier (Granja RG, Suzano, São Paulo, Brazil). When indicated, erythrocytes were treated with trypsin or papain under standardized conditions, followed by extensive washing to remove residual enzymes. All hemagglutination assays were performed in triplicate using three independent erythrocyte batches.

For the assay, 50 μL of lectin dilution was mixed with an equal volume (50 μL) of erythrocyte suspension and incubated for 60 min at room temperature. Hemagglutination was visually recorded, and one hemagglutinating unit (HU) was defined as the reciprocal of the highest lectin dilution showing complete agglutination under these conditions. Hemagglutinating activity was expressed as HU/mL, and specific activity was calculated as HU/mg protein.

Carbohydrate binding specificity was evaluated by hemagglutination inhibition assays.^15^ 2-fold serial dilutions of carbohydrates and glycoproteins (d-glucose, d-galactose, d-mannose, *N*-acetyl-d-glucosamine, *N*-acetyl-d-galactosamine, l-rhamnose, d-ribose, l-fucose, α-lactose, inosine, methyl-α-d-mannoside, fetuin, and mucin) were prepared in the same hemagglutinating activity buffer. Initial inhibitor concentrations were 100 mM for monosaccharides/disaccharides and 1 mg/mL for glycoproteins. Each inhibitor dilution was preincubated with lectin corresponding to 4 HU for 30 min at 37 °C, followed by addition of an equal volume of erythrocyte suspension and incubation for 60 min. The minimum inhibitory concentration (MIC) was defined as the lowest inhibitor concentration that completely prevented hemagglutination. All assays were performed in triplicate with controls (erythrocytes without lectin; lectin without sugar solutions; sugar solutions only).^11^

### SDS–PAGE, Glycoprotein Staining, and Size-Exclusion Chromatography

Purified EFusL was analyzed by SDS–polyacrylamide gel electrophoresis (SDS–PAGE) using 12.5% resolving gels under reducing conditions.^16^ Samples were prepared in SDS sample buffer containing 2-mercaptoethanol, heated at 95 °C for 5 min, and electrophoresed alongside a prestained molecular mass marker. Protein bands were visualized by Coomassie Brilliant Blue R-250 staining. The presence of carbohydrate moieties was qualitatively assessed by periodic acid–Schiff (PAS) staining.^17^ Total neutral carbohydrates were quantified by the phenol–sulfuric acid method.^18^ Glucose was used to generate the standard curve, and absorbance was measured at 490 nm. Protein concentration in crude extract, chromatographic fractions, and purified lectin was determined by the Bradford method^19^ using bovine serum albumin (BSA) as a standard, with absorbance measured at 595 nm.

The purity profile of EFusL under nondenaturing conditions was further evaluated by size-exclusion chromatography (SEC), following the same chromatographic conditions previously described. Briefly, 250 μL of the agarose–lactose purified fraction (4 mg/mL) was applied to a silica-based SEC column (BioSuite 250, 5 μm HR SEC) coupled to an ultraperformance liquid chromatography system (ACQUITY UPLC System, Waters). Chromatography was performed in isocratic mode using 100 mM sodium phosphate buffer, pH 7.0, containing 150 mM NaCl, at a flow rate of 400 μL/min. Protein elution was monitored by absorbance at 280 nm. The eluted fraction was collected, dialyzed against distilled water, lyophilized, and stored until further analysis.

### Physicochemical Stability and Divalent Cation Dependence

The influence of pH, temperature, and divalent cations on EFusL hemagglutinating activity was evaluated in three independent experiments (*n* = 3). For pH stability, lectin (1.0 mg/mL) was dialyzed for 24 h at 4 °C against 100 mM buffers containing 150 mM NaCl: sodium citrate (pH 4.0 and 6.0), sodium acetate (pH 5.0), sodium phosphate (pH 7.0), Tris–HCl (pH 8.0), and glycine–NaOH (pH 9.0 and 10.0). After dialysis, residual hemagglutinating activity was determined under the standard assay conditions described. Divalent cation dependence was assessed by dialyzing EFusL against 100 mM EDTA containing 150 mM NaCl for 24 h at 4 °C, followed by dialysis against 150 mM NaCl to remove excess EDTA. Residual hemagglutinating activity was measured before and after supplementation with CaCl_2_ and MnCl_2_ (5 mM each) for activity recovery. Thermal stability was evaluated by incubating the lectin solutions for 60 min at 30–100 °C. Samples were cooled to room temperature and centrifuged (9,000 × *g*, 10 min) to remove precipitated material prior to hemagglutination testing.^20^

### LC–MS/MS Sequencing, Genomic Amplification, and Phylogenetic Analysis

Protein bands corresponding to purified EFusL were excised from Coomassie-stained reducing SDS–PAGE gels and subjected to in-gel digestion essentially as described by ref 21, including destaining, reduction, alkylation, and proteolysis with proteolytic enzymes (trypsin, chymotrypsin, pepsin, Glu-C, and Asp-N) under protease-specific conditions. For peptide sequencing, 4.5 μL of the protease-digested EFusL sample was analyzed by RP-nanoUPLC on a C18 column (100 μm × 100 mm; nanoAcquity UPLC, Waters) coupled to an ESI-QUAD-TOF instrument (Micromass/Waters) at 0.600 μL/min using a 2–90% acetonitrile gradient in 0.1% formic acid over 20 min. MS/MS data were acquired in positive ion mode using data-dependent acquisition in “top three” mode with 60 s dynamic exclusion (real-time exclusion for endogenous peptides). Raw data were converted to MGF (without scan summing) in Mascot Distiller v2.3.2.0 and searched with Mascot v2.3.01 as an MS/MS Ion Search with an error-tolerant search strategy against custom lectin databases, using carbamidomethylation of cysteine as a fixed modification, oxidation of methionine as a variable modification, one missed cleavage, and ±1 Da tolerance for both peptide (precursor) and fragment ions; mass values were set to monoisotopic and instrument type to ESI-QUAD-TOF. MS-derived peptides were assembled into a consensus sequence and reconciled with the translated genomic sequence to resolve gaps/ambiguities and confirm the final 249-aa mature lectin chain. The mass spectrometry proteomics data have been deposited to the ProteomeXchange Consortium via the PRIDE partner repository with the dataset identifier PXD078105 and 10.6019/PXD078105.^22^

Primers targeting conserved regions of *Erythrina* lectin genes corresponding to the N- and C-terminal regions were designed based on the *Erythrina corallodendron* lectin mRNA sequence deposited in GenBank under accession number X52782.1. The primer sequences used for amplification were Erythrina-F, 5′-ACATGGCTACTTACAAGTTGTGC-3′, and Erythrina-R, 5′-TACATAGATGCTAACTGCGAGGTG-3′. Design and primer physicochemical characteristics were evaluated using NCBI Primer-BLAST.^23^ Genomic DNA was extracted from young leaves of *Erythrina fusca* using the CTAB method, quantified by spectrophotometry, treated with RNase A, and assessed by 0.7% agarose gel electrophoresis.^24^ PCR amplification was performed in a final volume of 25 μL containing 10× buffer, dNTPs, MgCl_2_, Erythrina-F and Erythrina-R primers, genomic DNA, nuclease-free water, and Taq DNA polymerase. The amplification program consisted of an initial denaturation at 94 °C for 5 min, followed by 35 cycles including annealing at 56 °C for 30 s and extension at 72 °C, with a final extension at 72 °C for 10 min. PCR products resulted in the amplification of a single visible band on the agarose gel,^25^ with an estimated size of 750 bp; the band was excised, and the amplicon was purified using the SV Gel and PCR Clean-Up Kit (Promega), following the manufacturer’s instructions. Purified amplicons were sequenced by Sanger sequencing directly using the EFusL-specific PCR primers.^26^ The resulting nucleotide sequence was translated and aligned with the MS/MS-derived peptide map to define the mature EFusL sequence and resolve uncovered or ambiguous regions.

Sequence alignments were performed using Clustal Omega,^27,28^ and graphical representations were prepared with ESPript3. Secondary structure prediction was carried out using PsiPred v4.0.^29,30^ When applicable, EFusL sequences and MS-derived peptides were compared with representative *Erythrina* lectins retrieved from public databases to evaluate residue conservation in the carbohydrate recognition domain and to support comparative analyses across homologues and isoforms.

### Molecular Modeling

Structural prediction was performed on the AlphaFold Server (https://alphafoldserver.com/), which uses the AlphaFold3 architecture. The method combines evolutionary information derived from multiple sequence alignments with physical-geometric constraints to directly predict the atomic coordinates of the protein.^31^ Predictions were conducted using the server’s standard parameters, including automatic MSA generation and internal ranking of the models, selecting the model with the highest confidence score. Structural quality was assessed based on the pLDDT (predicted Local Distance Difference Test) and the PAE (Predicted Aligned Error) matrix. pLDDT values above 90 were considered highly confident, while values below 70 indicated regions with lower reliability.^32^ Stereochemical validation was performed using MolProbity and the UCLA-DOE LAB server SAVES v6.1 (https://saves.mbi.ucla.edu/), which integrates different validation tools. The ERRAT program was used to analyze patterns of nonbonding interactions between atoms and identify potentially inconsistent regions in the structure.^33^ Verify3D was used to evaluate the compatibility between the three-dimensional structure and its primary structure, considering the structural environment of each residue.^34^ Stereochemical quality was also verified with PROCHECK through the analysis of dihedral angles and the distribution in the Ramachandran plot.^35^ Visualization and figure preparation were performed in PyMOL. Limitations are expected in flexible or poorly conserved regions, and the models were interpreted as computational predictions.^36,37^

### Molecular Docking with Galactosides

Molecular docking experiments were performed using GOLD v2025.1, considering the carbohydrate recognition domain (CRD) coordinates of EFusL as the receptor. The three-dimensional structures of the ligands galactose, *N*-acetyl-d-galactosamine, lactose, 2-α-l-fucosylactose, and *N*-acetyllactosamine were obtained from the PubChem database and previously prepared regarding protonation states and geometry. The binding site was defined from the geometric center of the CRD, including all residues within an 8 Å radius. Docking was conducted using the genetic algorithm implemented in the software, allowing conformational sampling of the ligands with standard program parameters.^38^ The scoring function used was ChemPLP, based on piecewise linear potentials and widely used for predicting binding modes in protein–ligand systems.^39^ The scoring functions consider empirical contributions associated with intermolecular interactions, such as hydrogen bonds, hydrophobic contacts, and steric complementarity, allowing for the relative comparison of affinity between ligands.^40^ For each compound, multiple poses were generated, and the one with the highest score was selected for analysis. Protein–ligand interactions were evaluated by structural inspection in PyMOL. For each ligand, 100 docking poses were generated using the genetic algorithm implemented in GOLD, with 100,000 genetic operations per run. Pose convergence was assessed by visual inspection and consistency of top-ranked solutions.

As a validation strategy, the same ligands were subjected to docking with homologous lectins, including ECL (*Erythrina cristagalli* lectin) and EcorL (*E. corallodendron* lectin), whose crystallographic structures complexed with carbohydrates are well characterized. For ECL, structures deposited in the Protein Data Bank include complexes with lactose (PDB ID: 1GZC), *N*-acetyllactosamine (PDB ID: 6AQ6), and 2-α-l-fucosyllactose (PDB ID: 1GZ9).^10,41^ For EcorL, crystallographic structures in complex with galactose, *N*-acetyl-d-galactosamine, and oligosaccharides are available under PDB codes 1AX1, 1AX2, 1AX0, and 1AXZ, demonstrating the conservation of the binding site and key interactions with carbohydrates.^9,42^ The comparison between the binding modes obtained for EFusL and those observed experimentally for ECL and EcorL was used as a qualitative validation criterion, considering the conservation of the residues involved in recognition and the similarity of the interaction patterns. Furthermore, molecular docking was performed with d-mannose, a monosaccharide not specifically recognized by lectins of the genus Erythrina. Analysis of the interactions was performed by visualization in PyMOL, Protein–Ligand Interaction Profiler (PLIP) server, and PoseView.^43–47^

### Toxicity Assays Using *Artemia* Nauplii and Human Cell Lines

EFusL toxicity toward *Artemia salina* nauplii was evaluated using seawater, which was filtered (0.45 μm). *A. salina* cysts were hatched at 1 g/L in seawater at 25 °C under constant aeration, and after 48 h only highly motile nauplii were selected for the assay. In 24-well plates, EFusL was tested over a serial dilution range (200 to 3.125 μg/mL) with 10 nauplii per well, using an appropriate final volume per well of 2 mL. Controls included seawater alone, lectin plus lactose to evaluate the participation of the CRD in the activity, and heat-denatured lectin.^48^ After 24 h, nauplii mortality was recorded, and nauplii were considered dead when no movement was observed after gentle agitation, and the *LC50* value was determined to be >200 μg/mL based on the maximum concentration tested.^49,50^

For cell-based assays, human cancer cell lines HeLa (ATCC CCL-2) and HT-1080 (ATCC CCL-121) and noncancerous human dermal fibroblasts NHDF (ATCC PCS-201-012) were maintained in DMEM supplemented with 10% (v/v) FBS and 1% (v/v) penicillin/streptomycin in T75 flasks at 37 °C in a humidified 5% CO_2_ atmosphere, with routine passaging at ∼80% confluence.^51^ Cell viability was assessed in 96-well plates using the PrestoBlue HS assay: cells were seeded at 5,000 cells/well in 100 μL and allowed to attach, then treated with EFusL diluted in complete DMEM to final concentrations of 125–7.8 μg/mL (serial dilutions). After 24, 48, and 72 h, culture medium was removed, and 100 μL of PrestoBlue HS (1× in PBS) was added.^52^ Plates were incubated for 1 h in the dark, and fluorescence was measured at λ_ex_ 560 nm/λ_em_ 590 nm using an Infinite F200 Pro microplate reader. Untreated cells were used as negative controls, and wells without cells were used to correct background signals.

## Results and Discussion

### Affinity-Purified EFusL Exhibits Strong Agglutination Activity and Galactoside Inhibition Profile

Hemagglutinating activity was readily detected in the *E. fusca* seed crude extract, and single-step lactose–agarose affinity chromatography yielded a lectin-enriched fraction with increased specific hemagglutinating activity relative to the crude material (Table 1; Figure 1A). SDS–PAGE analysis under reducing conditions showed closely migrating protein bands of approximately 29–31 kDa, whereas PAS staining confirmed the glycoprotein nature of EFusL (Figure 1B). This pattern is consistent with the subunit heterogeneity frequently reported for *Erythrina* seed lectins and other legume lectins, which commonly migrate as two closely spaced polypeptide bands under denaturing conditions (Table S1).^5,53,54^

**Table 1:** Purification of Lectin from *Erythrina fusca* Seeds

Fraction	^a^Total protein (mg/mL)	^b^Total H.U.	^c^Specific activity (H.U./mg)	Purification fold
Crude extract	22.5	128	5.7	1.0
PII agarose-lactose	2.1	64	30.5	5.4

a Protein content.

b Hemagglutinating activity was expressed in hemagglutinating units (H.U.).

c The specific activity was calculated as the ratio between hemagglutinating activity and protein content.

**Figure 1:**
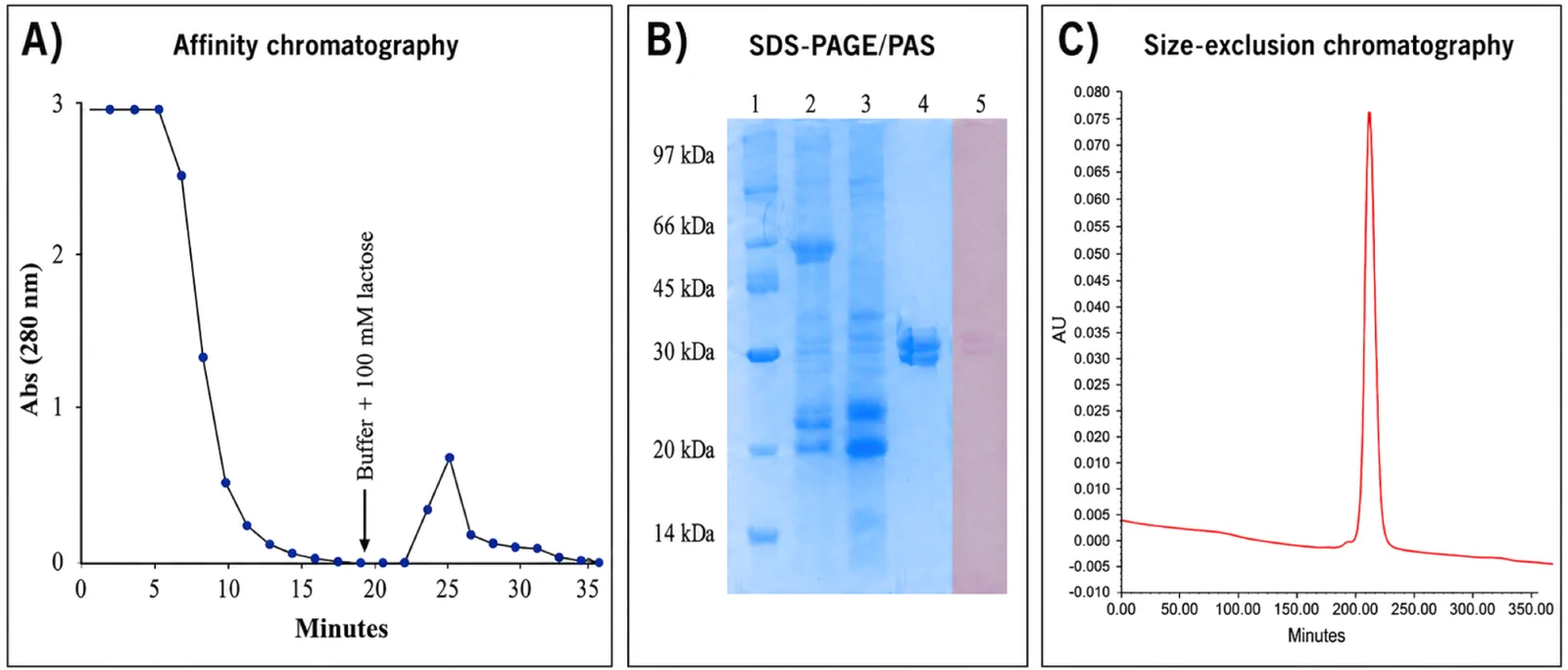
Purification of *Erythrina fusca* lectin. (A) Affinity chromatography in agarose-lactose gel. All fractions were collected and measured spectrophotometrically at 280 nm. (B) SDS–PAGE/PAS analysis: lane 1, TrueColor high range S2600 molecular marker; lane 2, crude extract; lane 3, nonretained fraction; lane 4, EFusL under reducing conditions; lane 5, EFusL stained with Schiff’s reagent. (C) Size-exclusion chromatography of purified EFusL, showing a single major peak consistent with a predominant native lectin species in solution.

To further evaluate the purity of the preparation under nondenaturing conditions, the agarose–lactose purified fraction was analyzed by SEC. The chromatographic profile showed a single major peak, supporting the predominance of one native EFusL species in solution (Figure 1C). Together, the SDS–PAGE/PAS and SEC profiles support the purity and homogeneity of the EFusL preparation used for subsequent functional and structural characterization.

Carbohydrate inhibition assays indicated a predominantly galactoside-binding profile: α-lactose was the most potent inhibitor, followed by d-galactose and *N*-acetyl-d-galactosamine, whereas l-fucose showed weaker but detectable inhibition (Figure 2A). This inhibition pattern is consistent with the canonical specificity reported for several *Erythrina* seed lectins, which typically recognize Gal/GalNAc- and lactose-related determinants.^5,11^ The structural basis underlying this conserved recognition profile and the sequence-level conservation of key carbohydrate-recognition residues are addressed in the structural/sequence analysis section below.

**Figure 2:**
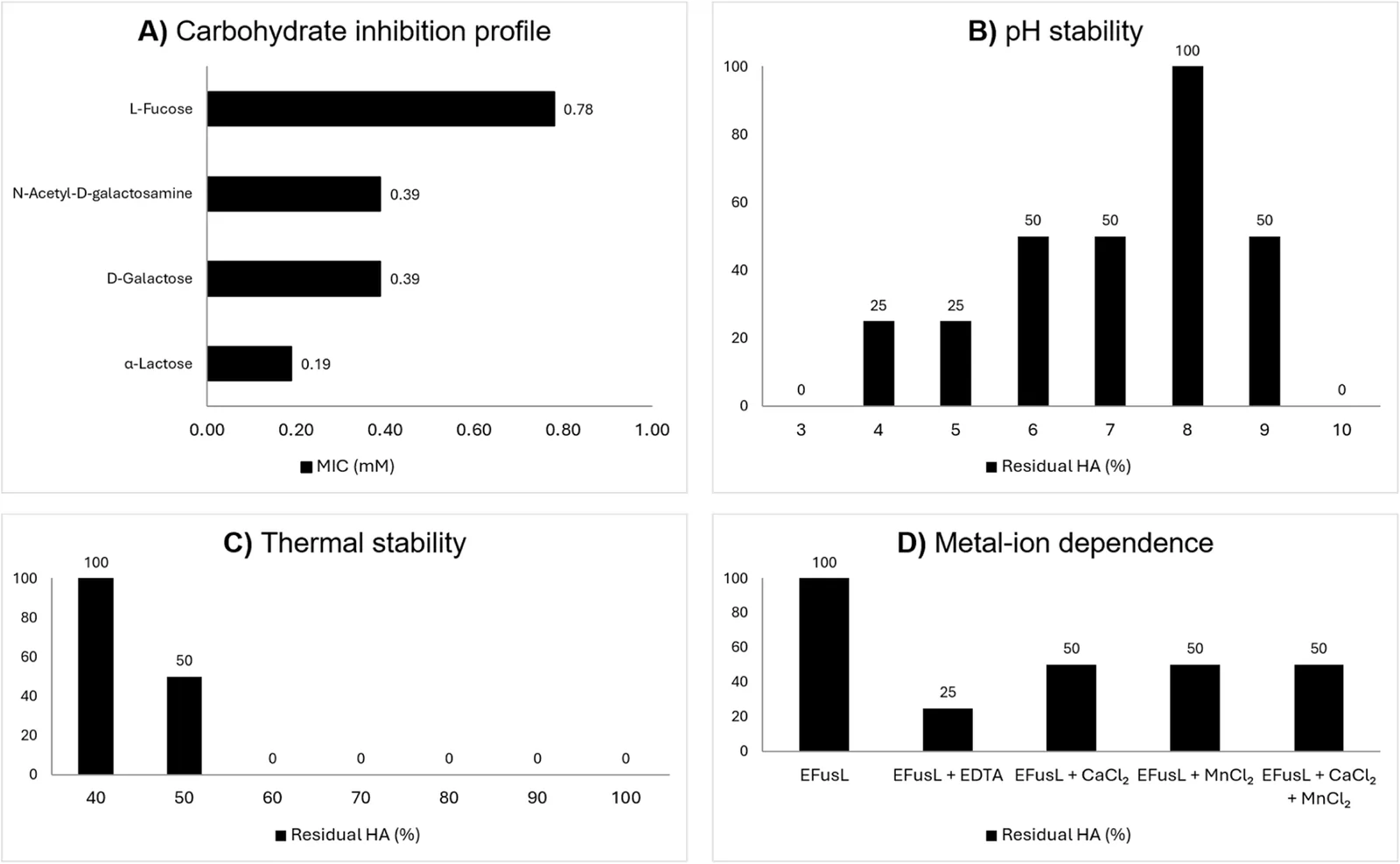
Functional and physicochemical properties of EFusL. (A) Minimum inhibitory concentration (MIC) values for carbohydrates that inhibited EFusL hemagglutinating activity. Lower MIC values indicate stronger inhibition. d-glucose, l-rhamnose, d-ribose, d-mannose, methyl-α-d-mannoside, inosine, and *N*-acetyl-d-glucosamine showed no inhibition up to 100 mM, whereas fetuin and mucin showed no inhibition up to 1 mg/mL. (B) Residual hemagglutinating activity after exposure to different pH values. (C) Residual hemagglutinating activity after incubation at different temperatures. (D) Effect of EDTA treatment and CaCl_2_ and/or MnCl_2_ supplementation on EFusL hemagglutinating activity. Residual activity is expressed relative to untreated EFusL, considered 100%. All assays were performed in independent triplicates (*n* = 3). Because hemagglutinating activity was determined by discrete 2-fold serial dilutions and identical endpoints were obtained in all replicates, SD values were zero and error bars are not visible.

### EFusL Exhibits Physicochemical Properties Consistent with Canonical *Erythrina* Lectins

Purified EFusL was confirmed as a glycoprotein with approximately 4.3% neutral carbohydrates, a value within the range commonly reported for glycosylated *Erythrina* lectins. EFusL displayed moderate thermal stability, maintaining full hemagglutinating activity up to 40 °C, retaining approximately 50% activity at 50 °C, and losing activity at higher temperatures under the conditions tested (Figure 2C). Regarding pH stability, EFusL showed maximal residual activity at pH 8.0 and retained a substantial fraction of activity near neutrality (pH 6.0–7.0) and at mildly basic pH (pH 9.0) (Figure 2B). The data regarding thermostability shows that this behavior is in line with the general thermal sensitivity reported for several *Erythrina* lectins, although more thermostable exceptions have been described in the genus (e.g., lectins remaining active at 60–70 °C) depending on the species and assay conditions. As for stability across different pH ranges, *Erythrina* lectins are reported to remain active across broader pH ranges (typically pH 6–10), while EFusL shows a narrower but still biologically relevant window of activity.^54–57^

Chelation experiments further suggested modulation by divalent cations: treatment with EDTA resulted in an approximately 75% reduction in hemagglutinating activity, and partial recovery was observed after supplementation with Ca^2+^, Mn^2+^, or Ca^2+^ plus Mn^2+^ (Figure 2D), consistent with divalent-cation-dependent behavior reported for several lectins of this genus.^54–57^ The partial restoration observed after cation supplementation suggests that metal coordination contributes to the maintenance of a functional carbohydrate-recognition site in EFusL, although additional structural studies are required to define the precise metal-binding geometry.

### Sequence Analysis Places EFusL Well within the Conserved *Erythrina* Lectin Subgroup

The complete primary structure of EFusL comprises 249 amino acids (Figure 3), which falls within the typical size range reported for mature *Erythrina* seed lectins (∼250–256 aa). This sequence assignment was supported by two complementary datasets: MS/MS-derived peptides and the translated nucleotide sequence obtained from the amplified lectin gene fragment. PCR amplification using *E. fusca* genomic DNA and primers designed from conserved regions of *Erythrina* lectin genes yielded a single amplicon of approximately 750 bp (Figure 3C-I). After purification and Sanger sequencing, the translated nucleotide sequence was aligned with peptides obtained by LC–MS/MS after digestion with trypsin, chymotrypsin, pepsin, Glu-C, and Asp-N (Figure 3C-II). The peptide coverage map shows that most of the mature EFusL sequence was directly supported by MS/MS-derived peptides, whereas regions not covered by MS/MS were assigned from the translated nucleotide sequence and further supported by conservation with phylogenetically related *Erythrina* lectins. These lectins are generally synthesized as secreted precursors containing an N-terminal signal peptide; for example, the *Erythrina corallodendron* lectin cDNA encodes a 281-residue precursor with an ∼25-residue signal peptide and an ∼256-residue mature chain.^58^ In addition, single-chain legume lectins, including *Erythrina* lectins, may undergo post-translational C-terminal trimming, which can reconcile minor discrepancies between cDNA-predicted and experimentally observed mature chains and provides a plausible explanation for small length offsets among highly homologous lectins.^59^

**Figure 3:**
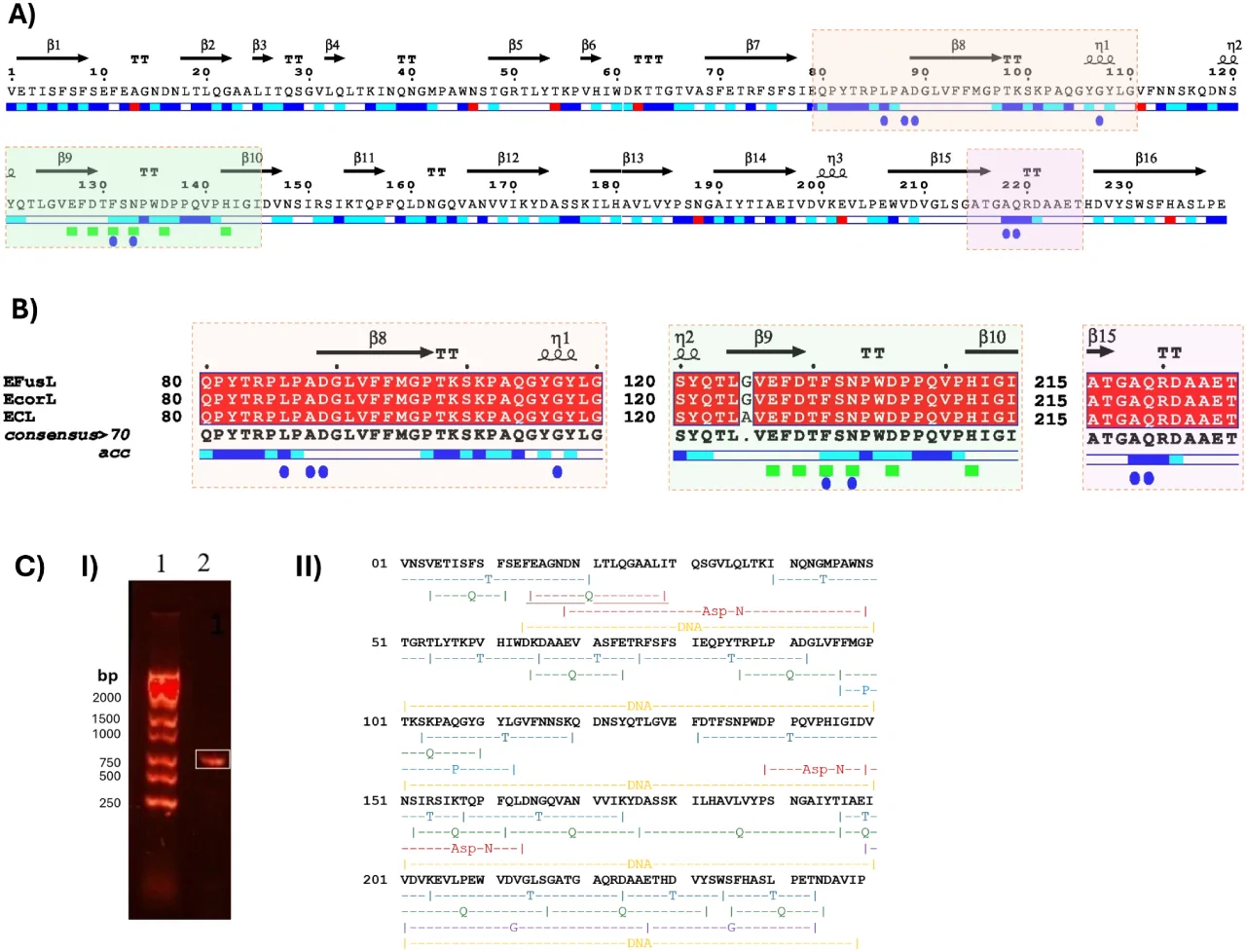
Secondary-structure map and alignment of EFusL functional regions. (A) Secondary-structure annotation of the mature EFusL sequence showing β-strands, β-turns (TT), short 3_10_-helical segments (η), and relative solvent accessibility (acc). The three colored boxes indicate the sequence regions enlarged in panel B. (B) Alignment of the selected CRD/MBS-containing regions of EFusL with EcorL and ECL. The colored blocks correspond to the boxed regions shown in panel A. Identical residues are highlighted in red; consensus >70 indicates positions conserved in >70% of the aligned sequences, and acc indicates relative solvent accessibility. Blue circles mark carbohydrate-recognition domain (CRD) residues, and green squares mark Ca^2+^/Mn^2+^ metal-binding site (MBS) residues. Residue numbering refers to the mature EFusL sequence. (C-I) Agarose gel electrophoresis of the PCR product corresponding to the *E. fusca* lectin gene amplicon. Lane 1, molecular marker; lane 2, amplified EFusL gene fragment. (C-II) Peptide coverage map of EFusL showing MS/MS-derived peptides obtained after digestion with different proteases and their alignment with the translated nucleotide sequence. Residue numbering refers to the EFusL sequence.

BLAST searches highlighted strong conservation within the genus, showing 96.8% identity to the lactose-specific lectin from *E. corallodendron* (UniProt P16404) and 95.4% identity to the lectin from *E. cristagalli* (UniProt P83410). Consistently, multiple sequence alignment revealed extensive conservation across EFusL and related *Erythrina* lectins, including residues discussed as part of the carbohydrate-recognition domain (e.g., Ala88, Asp89, and Gly107; Figure S1). The terminal segments of EFusL also match the conserved pattern reported for *Erythrina* lectins, with N-terminal “VETISFSFSEFEAGN” and C-terminal “AAETHDVYSWSFHASLPETN” sequences (Table S2), supporting its placement among conserved galactoside-binding *Erythrina* lectins.

A conserved yet functionally relevant feature in this clade is *N*-glycosylation. Sequence alignment reveals that EFusL shares a potential canonical *N*-glycosylation sequon (N-X-T), which is strictly conserved with the Asn17–Leu18–Thr19 site experimentally mapped in *E. corallodendron* lectin. In the latter, structural studies linked a bulky *N*-glycan to the distinctive (“handshake”) mode of dimerization described for this protein.^58,59^ Similarly, in *E. cristagalli* lectin, an *N*-linked oligosaccharide at Asn113 contributes to intermolecular contacts, suggesting that glycan occupancy may modulate quaternary association and surface properties across the genus.^60^ However, since direct glycopeptide MS/MS evidence or enzymatic deglycosylation (e.g., using PNGase F or Endo H) was not performed for EFusL in this study, its precise glycosylation site occupancy, glycan structure, and definitive functional impact remain undetermined. Therefore, while EFusL is firmly established here as a glycoprotein, further structural studies are required to fully characterize its glycan framework.

*Erythrina* lectins illustrate how high sequence identity can coexist with meaningful differences in ligand accommodation. Structure-guided mutagenesis of *E. corallodendron* lectin identified key combining-site determinants for galactoside recognition—Asp89, Asn133, and an aromatic stacking residue (Phe131 in EcorL)—while other positions can tune recognition of LacNAc-related epitopes.^60^ Consistently, only a small set of amino acid differences has been reported between ECL and EcorL, yet these closely related lectins display measurable differences in binding behavior, emphasizing that high identity does not preclude biologically relevant specificity shifts.^61^ Genus-wide comparisons further reinforce that primary structure conservation extends to the N-terminus, and comparative inhibition analyses support a broadly conserved galactoside/LacNAc-centered recognition trend across *Erythrina* lectins,^11^ providing additional context for positioning EFusL within this canonical subgroup.

### Structural Modeling and Docking Analyses Support a Conserved Galactoside-Recognition Architecture in EFusL

Figure 3 maps the secondary-structure elements of the legume lectin fold onto the EFusL sequence alignment, revealing an architecture dominated by β-strands (β1−β16) and turns, consistent with the conserved *jellyroll*/β-sandwich scaffold reported for *Erythrina* lectins by crystallography.^9,62^ The predicted structural model of EFusL resulted in an architecture with high reliability. The model presented a pTM (predicted TM-score) of 0.97, indicating high accuracy in the overall arrangement of the protein, while all residues exhibited pLDDT values >90, consistent with high local prediction confidence.^31^

Structural validation of the EFusL model confirmed a high stereochemical quality and environmental compatibility. The ERRAT value (91.34) and Verify3D score (91.43% compatibility) exceeded the generally accepted thresholds for reliable models (>80%).^33,34^ Furthermore, the Ramachandran plot obtained by PROCHECK indicated that 91.7% of the residues are located in more favored regions and 8.3% in additionally allowed regions, with no residues in generously allowed or unfavored regions, evidencing adequate conformational geometry of the protein.^35^ Additionally, the model showed an absence of inadequate steric contacts and 96.6% of the planar groups within acceptable limits, which are parameters compatible with high-quality structural models.

EFusL presented a structural architecture compatible with classic legume lectins, characterized by the jellyroll motif or β-sandwich folding, formed by two antiparallel β-sheets organized in compact structural planes, one composed of seven curved β-strands and the other of six more extended β-strands, connected by a loop (Figure 4A). This structural pattern remains highly conserved among plant lectins, acting as a conformational scaffold for the carbohydrate recognition domain (CRD) and directly contributing to carbohydrate binding specificity.^2,3^ The observed organization showed high similarity to the lectins of *Erythrina cristagalli* and *Erythrina corallodendron*, both described with a conserved β-sandwich core and maintenance of residues involved in galactoside recognition, reinforcing structural conservation within the genus *Erythrina*.^10,41,62,63^ Structural superposition analyses further confirmed this similarity, revealing low RMSD values between EFusL and ECL (0.184 Å over 209 Cα atoms) and between EFusL and EcorL (0.169 Å over 209 Cα atoms), indicating a highly conserved three-dimensional arrangement among these lectins. This structural arrangement is associated with the conformational stability and biological functionality of these proteins, despite the variability observed in carbohydrate specificity within this family.^3^

**Figure 4:**
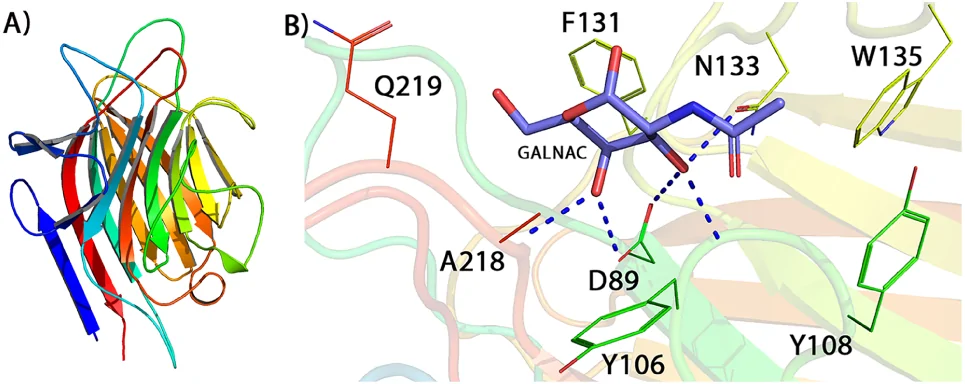
Structural model and carbohydrate recognition domain of EFusL. (A) Predicted three-dimensional structure of EFusL represented in a cartoon model, highlighting the β-sheet-rich folding pattern characteristic of legume lectins. (B) Detailed view of the carbohydrate recognition domain of EFusL complexed with *N*-acetyl-d-galactosamine (GalNAc). The amino acid residues involved in ligand recognition are represented in a line model, and GalNAc in stick form (with carbons in purple), while hydrogen interactions are indicated by dashed blue lines.

Molecular docking analysis demonstrated that the lectin from *Erythrina fusca* (EFusL) exhibited favorable binding affinity toward all evaluated carbohydrates, with docking scores ranging from −27.29 to −43.98 (Table 2). Among the tested ligands, fucosyllactose displayed the most favorable interaction with EFusL (−43.98), followed by *N*-acetyllactosamine (−35.04), GalNAc (−32.58), lactose (−31.83), and galactose (−27.29). When compared with lectins from *Erythrina cristagalli* (ECL) and *Erythrina corallodendron* (EcorL), EFusL exhibited a similar ligand preference profile, although with slightly less favorable binding. In all evaluated lectins, the more complex oligosaccharides, particularly fucosyllactose and *N*-acetyllactosamine, showed lower binding energies, suggesting enhanced stabilization of the protein–ligand complex due to the greater number of intermolecular contacts. This pattern indicates that the increased interaction surface and availability of hydroxyl groups favor the establishment of more extensive hydrogen-bonding networks and complementary hydrophobic contacts within the carbohydrate recognition domain.

**Table 2:** Results of Molecular Docking Analyses between the Lectins EFusL, ECL, and EcorL and Different Carbohydrates (Score Values Are in Arbitrary Units)

Carbohydrate	EFusL	ECL	EcorL
*N*-acetyl-d-galactosamine	–32.58	–35.31	–35.83
d-galactose	–27.29	–31.34	–31.54
lactose	–31.83	–37.35	–37.10
2-α-l-fucosylactose	–43.98	–47.79	–48.31
*N*-acetyllactosamine	–35.04	–39.97	–40.49
Tn-antigen	–36.72	–40.31	–41.79
TF-antigen	–32.48	–38.12	–38.33
sTn-antigen	–24.84	–28.01	–28.63
d-mannose	–23.59	–26.82	–26.87

Structural analysis of the docking-derived complexes revealed a conserved molecular recognition pattern predominantly mediated by hydrogen bonds involving residues Asp89, Gly107, Asn133, Ala218, and Gln219 (Figure 4B; Figure S2A). In the EFusL–GAL complex, the OD2 atom of Asp89 interacted with the O_4_ atom of galactose, exhibiting an H–A (hydrogen–acceptor) distance of 2.29 Å, a D–A (donor–acceptor) distance of 3.21 Å, and a bond angle of 158.84°, indicating a geometrically favorable interaction. Gly107 interacted through its backbone N atom with the O_3_ atom of GAL (H–A = 2.27 Å; D–A = 3.11 Å; angle = 139.11°), whereas Asn133 established two distinct hydrogen bonds involving the OD1/N atoms of the residue and the O_2_ and O_3_ hydroxyl groups of the ligand, including a strong interaction with O_2_ (H–A = 2.04 Å; D–A = 2.91 Å; angle = 150.36°). The Ala218 contributed through its backbone N atom interacting with the O_4_ atom of GAL (H–A = 2.06 Å; D–A = 3.04 Å; angle = 160.54°), while Gln219 formed an interaction between its side-chain N atom and the O_6_ atom of galactose (H–A = 1.92 Å; D–A = 2.87 Å; angle = 155.35°). The GalNAc and lactose complexes maintained this conserved interaction network, indicating that these residues compose the primary galactoside recognition site of EFusL.

The lack of efficient recognition of mannosides by EFusL likely arises from stereochemical differences between galactose and mannose, particularly in the orientation of the hydroxyl group at the C4 carbon. While galactose displays an axial configuration at this position, favoring the hydrogen-bonding network established with Asp89, Gly107, Asn133, Ala218, and Gln219, mannose exhibits an equatorial configuration that is incompatible with the geometry of the EFusL carbohydrate-recognition site. This behavior is characteristic of galactoside-specific lectins from the genus *Erythrina*.^2,64^ Supporting this hypothesis, molecular docking performed with d-mannose yielded a lower affinity score (−23.59) compared to d-galactose (−27.29), indicating reduced stability of the EFusL–mannose complex. This decrease in affinity was mainly associated with the loss of hydrogen bonds involving the Asn133 residue, considered one of the key structural determinants for galactoside recognition at the EFusL binding site.

However, ligand-specific differences were also observed. The GalNAc complex (Figure S2B) exhibited an additional hydrophobic interaction involving Trp135 and the N-acetyl moiety of the ligand, with a distance of 3.54 Å, suggesting a role in the differential stabilization of this carbohydrate. In contrast, lactose established a larger number of hydrogen bonds, particularly involving Asn133 and Gln219, likely due to the additional hydroxyl groups present in the disaccharide structure. Collectively, these findings demonstrate that the ligand recognition specificity of EFusL is governed by a conserved network of polar interactions primarily directed toward the O_2_, O_3_, O_4_, and O_6_ hydroxyl groups of galactosides, complemented by additional contacts associated with the distinct structural features of each ligand.

The results obtained for EFusL corroborate recent structural descriptions of legume lectins specific for galactosides, in which molecular recognition is mediated mainly by hydrogen bonds involving the O_3_, O_4_, and O_6_ hydroxyl groups of carbohydrates, associated with complementary hydrophobic contacts.^2,3^ The greater number of interactions observed for lactose, fucosyllactose, and *N*-acetyllactosamine (Figure S2C–E) suggests greater stabilization of the protein–ligand complex due to the expansion of the contact surface and the network of polar interactions. Furthermore, the structural overlap of the EFusL-carbohydrate complexes with the crystallographic structures of ECL and EcorL complexed with the same ligands demonstrated high conservation of the interaction mode in the carbohydrate recognition site (Figure 5), reinforcing the structural and functional preservation of the CRD domain among lectins of the genus *Erythrina*.

**Figure 5:**
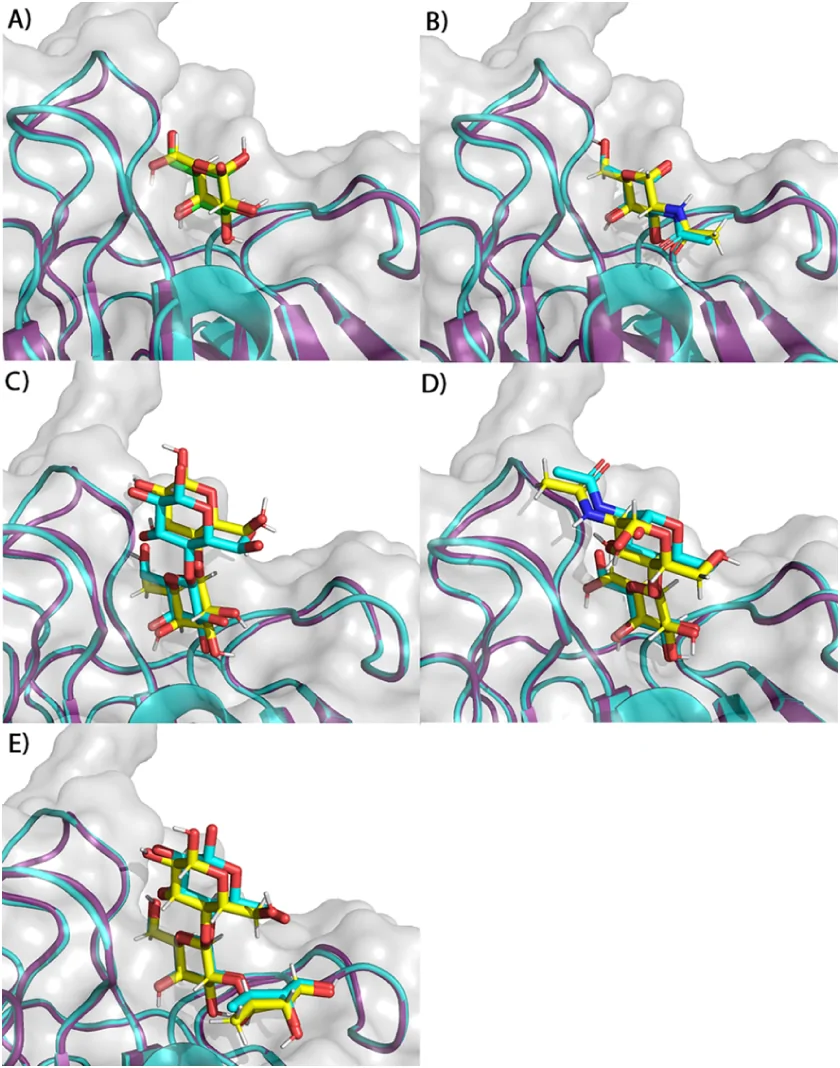
Structural superposition of ligand-bound EFusL with similar lectins for binding mode comparison. The structural model of EFusL is represented in purple, while the reference lectins obtained from the Protein data Bank (PDB) are represented in blue. Carbohydrates are represented in stick models, with the carbon atoms in yellow corresponding to the ligands obtained from the molecular docking of EFusL and, in blue, to the ligands present in the crystallographic structures. EFusL was superposed with (A) galactose-bound EcorL (PDB: 1AXZ), (B) *N*-acetylgalactosamine-bound EcorL (PDB: 1AX0), (C) lactose-bound ECL (PDB: 1GZC), (D) *N*-acetyllactosamine-bound EcorL (PDB: 1AX2), and (E) 2′-α-l-fucosyllactose-bound ECL (PDB: 1GZ9).

Regarding molecular docking with galactoside-based tumor-associated antigens, EFusL showed distinct interaction profiles for Tn, TF, and sTn, directly reflecting the observed affinity scores and the accessibility of the Gal/GalNAc recognition core within the CRD (Figure S3). The Tn antigen showed the best docking score (−36.72), indicating higher stability of the protein–ligand complex. In the EFusL–Tn complex, interactions mainly involved residues Asp89, Gly107, Asn133, Ala218, and Gln219, along with hydrophobic contacts with Tyr108 and Trp135. Asp89 formed a hydrogen bond with the O_2_ atom of GalNAc, with a D–A distance of 3.11 Å and an angle of 168.61°. Gly107 interacted with the ligand O_2_ atom through the backbone nitrogen, showing a D–A distance of 2.94 Å and an angle of 143.38°. Asn133 established two interactions with the O_2_ and O_3_ atoms of GalNAc, with D–A distances of 2.88 Å and 4.04 Å and angles of 154.31° and 119.19°, respectively. Ala218 interacted with the O_3_ atom of the carbohydrate (D–A = 2.91 Å; angle = 159.80°), while Gln219 formed a hydrogen bond with another O_3_ atom of the ligand (D–A = 2.82 Å; angle = 158.29°). Hydrophobic contacts with Tyr108 and Trp135 were observed at distances of 3.56 Å and 3.57 Å, respectively. These interactions indicate that Tn recognition occurs mainly through the hydroxyl groups of the GalNAc ring, preserving the characteristic binding pattern of galactoside-specific lectins. The TF antigen showed a docking score of −32.48 and retained part of the interaction network associated with galactoside recognition by EFusL. In the EFusL–TF complex, interactions involved residues Asp89, Gly107, Asn133, and Ala218. Recognition occurred predominantly through the terminal galactose ring of the disaccharide. Asp89 formed a hydrogen bond with the O_2_ atom of the terminal galactose (D–A = 2.95 Å; angle = 157.77°), while Gly107 interacted with the O_3_ atom of the ligand through the backbone nitrogen (D–A = 3.17 Å; angle = 147.62°). Asn133 established two interactions with the O_2_ and O_3_ atoms of the terminal galactose, with D–A distances of 2.87 Å and 4.08 Å and angles of 158.96° and 154.41°, respectively. Ala218 also contributed to complex stabilization through interaction between the backbone nitrogen and the O_3_ atom of the terminal carbohydrate (D–A = 3.03 Å; angle = 156.20°). These results indicate that TF recognition is primarily mediated by the terminal galactose residue, maintaining the typical binding profile of galactoside-specific lectins. In contrast, the sTn antigen showed the lowest docking score (−24.84), indicating lower stability of the EFusL–sTn complex. Unlike Tn and TF, recognition occurred predominantly through the terminal sialic acid residue, while the GalNAc ring remained less accessible to the lectin binding site. The sialyl group likely caused partial steric hindrance of the GalNAc core, impairing formation of the conserved hydrogen-bond network involved in galactoside recognition by EFusL. As a result, the EFusL–sTn complex displayed substantially weaker interactions compared to Tn and TF.

Tn, TF, and sTn antigens are recognized as biomarkers associated with aberrant glycosylation in tumor cells, being related to tumor progression, invasion, and poorer prognosis.^65,66^ In gliomas and glioblastomas, alterations in the O-glycosylation profile and increased expression of TF/T-antigen-type antigens have already been associated with progression-free survival and tumor aggressiveness.^67^ In this context, the high affinity observed between EFusL and Tn and TF antigens suggests potential application of this lectin in the recognition of tumor glycophenotypes associated with gliomas and other solid tumors. This affinity is directly associated with the lectin’s nominal specificity for the core carbohydrates (GalNAc and Gal) that compose these antigenic determinants. This binding profile finds solid structural precedent in other Gal/GalNAc-specific legume lectins, such as *Vatairea macrocarpa* lectin (VML), whose crystal structure complexed with the Tn antigen (PDB: 4XTP) demonstrates that these tumor-associated sugars accommodate into the canonical CRD. Remarkably, EFusL shares the strictly conserved core triad driving primary carbohydrate coordination (Asp89, Gly107, and Asn133, corresponding to Asp87, Gly105, and Asn129 in VML). While peripheral loop residues exhibit variations characteristic of each genus (such as Phe131, Gly217, Ala218, and Gln219 in EFusL compared to Gly212, Leu213, Ser214, and His217 in VML), the foundational framework required to anchor these tumor-associated antigens remains structurally preserved. Nevertheless, antigen binding does not necessarily imply a cytotoxic effect. This behavior contrasts with mannose/glucose-specific legume lectins, which are known to promote strong glioma cytotoxicity (for review, see Leal et al., 2025 ^68^). Furthermore, given that direct binding assays were not performed, the observed lack of toxicity may stem from the low expression of compatible carbohydrate receptors in these specific cell models, rather than a limitation in glycan accessibility, a hypothesis that warrants further investigation.

### EFusL Toxicity in *Artemia salina* and Human Cells

In the *Artemia salina* lethality assay, EFusL did not induce mortality at the tested concentrations and time points, indicating that its LC50 is above the evaluated range. In cell viability assays, EFusL did not significantly affect NHDF viability, whereas moderate reductions in HeLa and HT-1080 viability were observed under selected concentration- and time-dependent conditions (Figure 6; Figure S4). These results are compatible with the hypothesis that EFusL has limited access to a sufficient density of functional cell-surface ligands in these models. EFusL belongs to the *Erythrina* lectin group, whose carbohydrate recognition is directed toward galactoside-containing structures, including Gal/GalNAc and, more prominently for ECL-like lectins, terminal type-II LacNAc motifs such as Galβ1–4GlcNAc.^11^ However, biological effects of lectins depend not only on nominal sugar specificity but also on ligand density, accessibility, multivalent engagement, receptor clustering, internalization, and downstream cellular responses. The complete dataset, including the 72 h time point, is provided in the Supporting Information (Figure S4).

**Figure 6:**
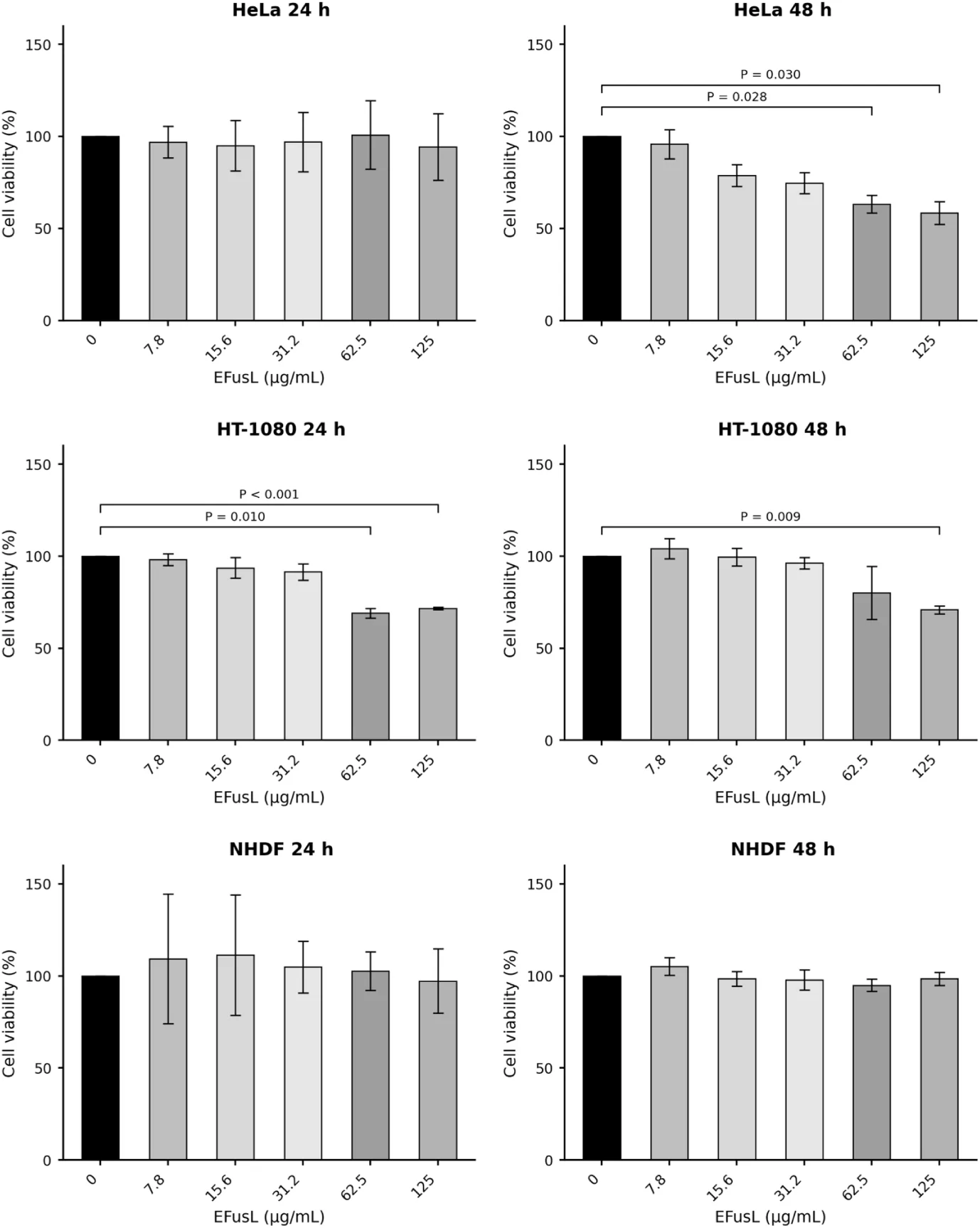
Effect of EFusL on the viability of human cell lines at 24 and 48 h. HeLa, HT-1080, and NHDF cells were treated with EFusL at concentrations ranging from 7.8 to 125 μg/mL for 24 and 48 h. Cell viability was assessed using the PrestoBlue HS assay and normalized to untreated cells, which were set to 100% viability within each independent experiment. Data are expressed as mean ± SD of three independent biological experiments, performed with four technical replicates per condition. Statistical comparisons were performed against 100% viability, with Holm-adjusted *p*-values indicated when significant. The complete dataset, including the 72 h time point, is shown in Figure S4.

In *Artemia*, the absence of lethality may, therefore, reflect insufficient presentation or accessibility of terminal β-galactoside/LacNAc-like motifs required for stable multivalent EFusL engagement. Available glycosphingolipid analyses in brine shrimp emphasize complex and highly fucosylated structures, including fucosylated LacdiNAc-like sequences and α-GlcNAc-terminated motifs, which may not optimally support binding and cross-linking by an ECL/EFusL-like galactoside-binding lectin.^69^ This interpretation is consistent with studies on other legume lectins showing that toxicity in *A. salina* can be carbohydrate-recognition dependent, as carbohydrate blocking of the CRD reduces lectin binding to the nauplii digestive tract and attenuates lethality.^70^

For the human cell lines, the limited and cell-type-dependent effects of EFusL are also consistent with the poor functional exposure of EFusL-reactive ligands. ECL/ECA-type binding is favored by unmodified terminal LacNAc and can be reduced when these motifs are capped or modified, particularly by sialylation.^71^ Thus, even if Gal/LacNAc-containing glycans are present on HeLa, HT-1080, or NHDF cells, they may be insufficiently exposed, insufficiently clustered, or unable to trigger the internalization or signaling events required for cytotoxicity. Therefore, the limited cytotoxic response to EFusL should be interpreted as compatible with limited functional ligand accessibility rather than as definitive proof of glycan mismatch.

This interpretation is also consistent with reports for other lectin–cell systems. Some plant lectins exhibit cytotoxicity closely associated with ligand availability and glycan-dependent binding, such as mannose-specific lectins whose effects differ among HeLa, HT-1080, and NHDF cells according to surface glycan presentation.^72^ Conversely, other lectins tested in similar concentration ranges show minimal cytotoxicity toward these same cell models, reinforcing that cellular responses depend critically on the combined effects of fine carbohydrate specificity, ligand density and presentation, multivalent cross-linking, internalization, and activation of downstream pathways such as apoptosis or autophagy, rather than simply on the presence of the lectin in the culture medium.^72,73^

## Conclusion

EFusL emerges as a structurally conserved member of the *Erythrina* lectin group that can be readily isolated by single-step lactose-affinity chromatography and retains the characteristic galactoside/LacNAc-centered inhibition profile described for related lectins. Its physicochemical behavior, including moderate thermal stability, pH-dependent hemagglutinating activity, and sensitivity to metal chelation, remains consistent with properties commonly reported across the genus. Sequence analysis and structural modeling further support preservation of the canonical legume lectin β-sandwich architecture and conservation of the carbohydrate-recognition framework shared with EcorL and ECL. Despite maintaining these classical lectin features, EFusL did not induce lethality in *Artemia salina* and did not significantly affect NHDF viability, although moderate reductions in HeLa and HT-1080 viability were observed under selected conditions. These findings indicate that glycan accessibility and presentation may be limiting factors for productive biological interactions in these systems. Altogether, this work expands the comparative framework available for understanding structure–recognition relationships within galactoside-binding legume lectins.

## Supplementary Material



## Data Availability

The mass spectrometry proteomics data underlying this study have been deposited to the ProteomeXchange Consortium via the PRIDE partner repository with the dataset identifier PXD078105. Other biochemical and biological data supporting the findings of this study are available from the corresponding author upon reasonable request. No registration or data use agreement is required for access.

## Notes

Full EFusL sequence alignment and secondary-structure annotation (Figure S1); two-dimensional interaction diagrams of EFusL with tumor-associated O-glycan antigens and galactoside ligands (Figures S2 and S3); complete cell viability dataset for HeLa, HT-1080, and NHDF cells at 24, 48, and 72 h (Figure S4); and comparative tables of the quaternary structure and N- and C-terminal sequences of *Erythrina* lectins (Tables S1 and S2) (PDF)
