# Supplementary material for: Functional and Structural Characterization of the Erythrina fusca Seed Lectin EFusL: A GalNAc-Binding Lectin with Specificity for Tumor-Associated O‑Glycans
Source: ACS Omega. 2026 Aug 18;11(34):51677–89. doi: 10.1021/acsomega.6c05946 (PMC13625548; doi:10.1021/acsomega.6c05946)
Supplement: Supplementary file 1 [file ao6c05946_si_001.pdf]

Functional and structural characterization of the *Erythrina fusca* seed lectin EFusL: a GalNAc-binding lectin with specificity for tumor-associated O-glycans

Messias V. Oliveira[1], Francisco W. V. Martins[1], Clara Suarez[1], Valeria M. S. Ferreira[1], Vanir R. Pinto-Junior[1], Vinicius J. S. Osterne[1], Luiz A. G. Souza[2], Els J. M. Van Damme[3], Rodrigo B. Leal[4], Benildo S. Cavada[1,*] and Kyria S. Nascimento[1,*]

[1]Federal University of Ceara, Department of Biochemistry and Molecular Biology, Pici Campus Prof. Prisco Bezerra, S/N, Pici, 60.455-970, Fortaleza, Brazil

[2]National Institute for Research in the Amazon, Department of Agronomic Sciences, 2936 Andre Araujo Avenue, Petropolis, 69067-375, Manaus, Brazil

[3]Ghent University, Laboratory of Biochemistry and Glycobiology, Department of Biotechnology, Proeftuinstraat 86, 9000, Ghent, Belgium

[4]Federal University of Santa Catarina, Department of Biochemistry, Biological Sciences Center, Block G, 2nd Floor, Room 219, 88037-000, Florianópolis, Brazil

*Corresponding authors: K.S.N. (kyriasantiago@ufc.br); B.S.C. (bscavada@ufc.br)

Figure S1. Sequence alignment and secondary-structure annotation of EFusL. The mature EFusL sequence determined in this study was aligned with representative Erythrina lectins. The multiple sequence alignment was rendered with ESPript: identical residues are shaded in red and conserved substitutions are boxed. Secondary-structure elements of the canonical legume lectin fold are mapped above the alignment, including β-strands (βA–βP), turns (TT), and short 3_10 helices (ηA–ηC). Residue numbering refers to EFusL, and the "consensus >70" track highlights positions conserved in ≥70% of the aligned sequences.

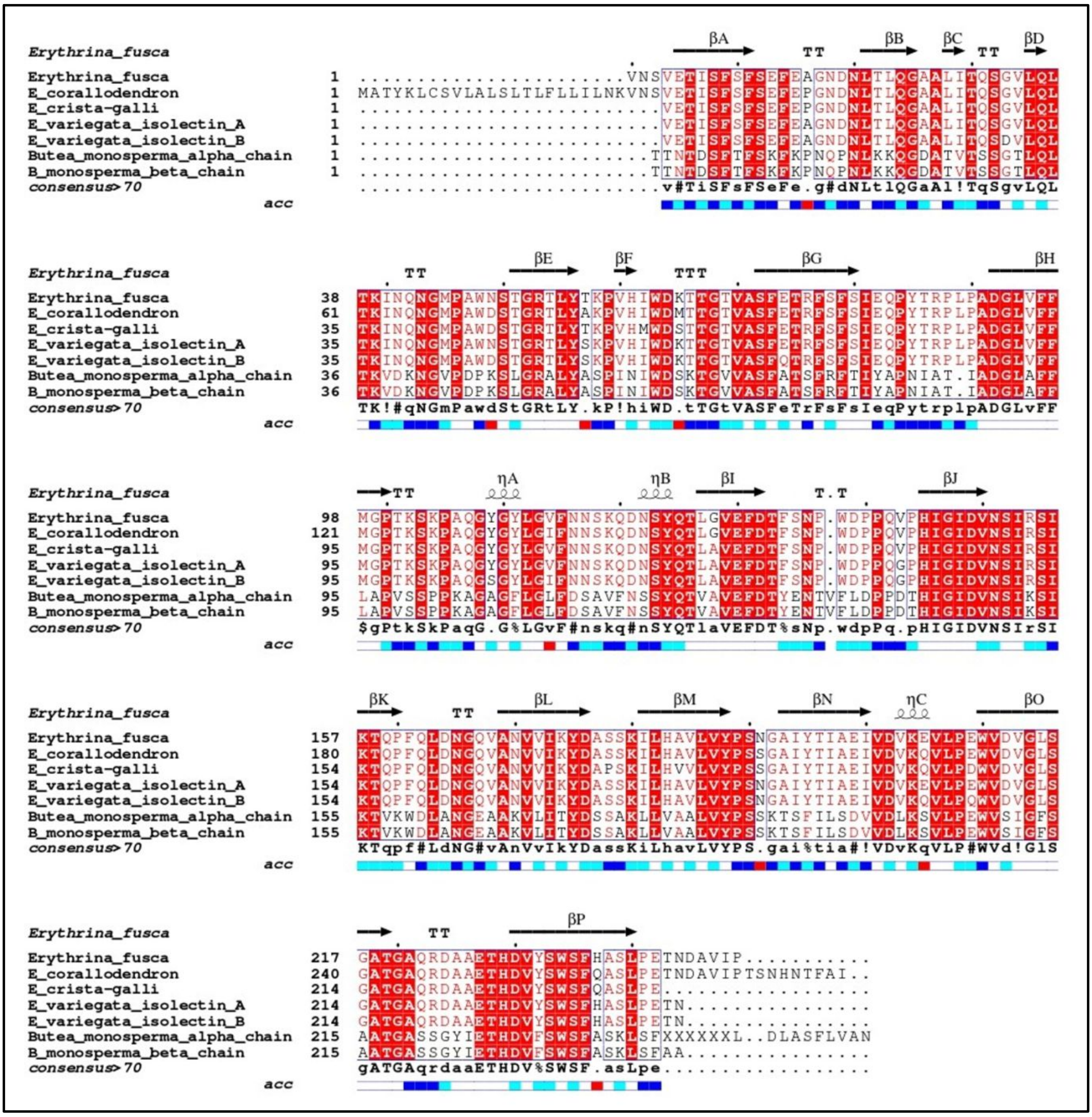

Figure S2. Two-dimensional interaction diagrams of EFusL with galactoside ligands evaluated by molecular docking. (A) D-galactose. (B) N-acetyl-D-galactosamine. (C) Lactose. (D) N-acetyllactosamine. (E) 2′-α-L-fucosyllactose. Dashed lines indicate predicted hydrogen-bond interactions between the ligands and EFusL residues. The main interacting residues are labeled in each panel, including Asp89, Gly107, Asn133, Ala218, Gln219, and, when present, Tyr108. These interaction maps support the involvement of the conserved EFusL carbohydrate-recognition region in galactoside binding.

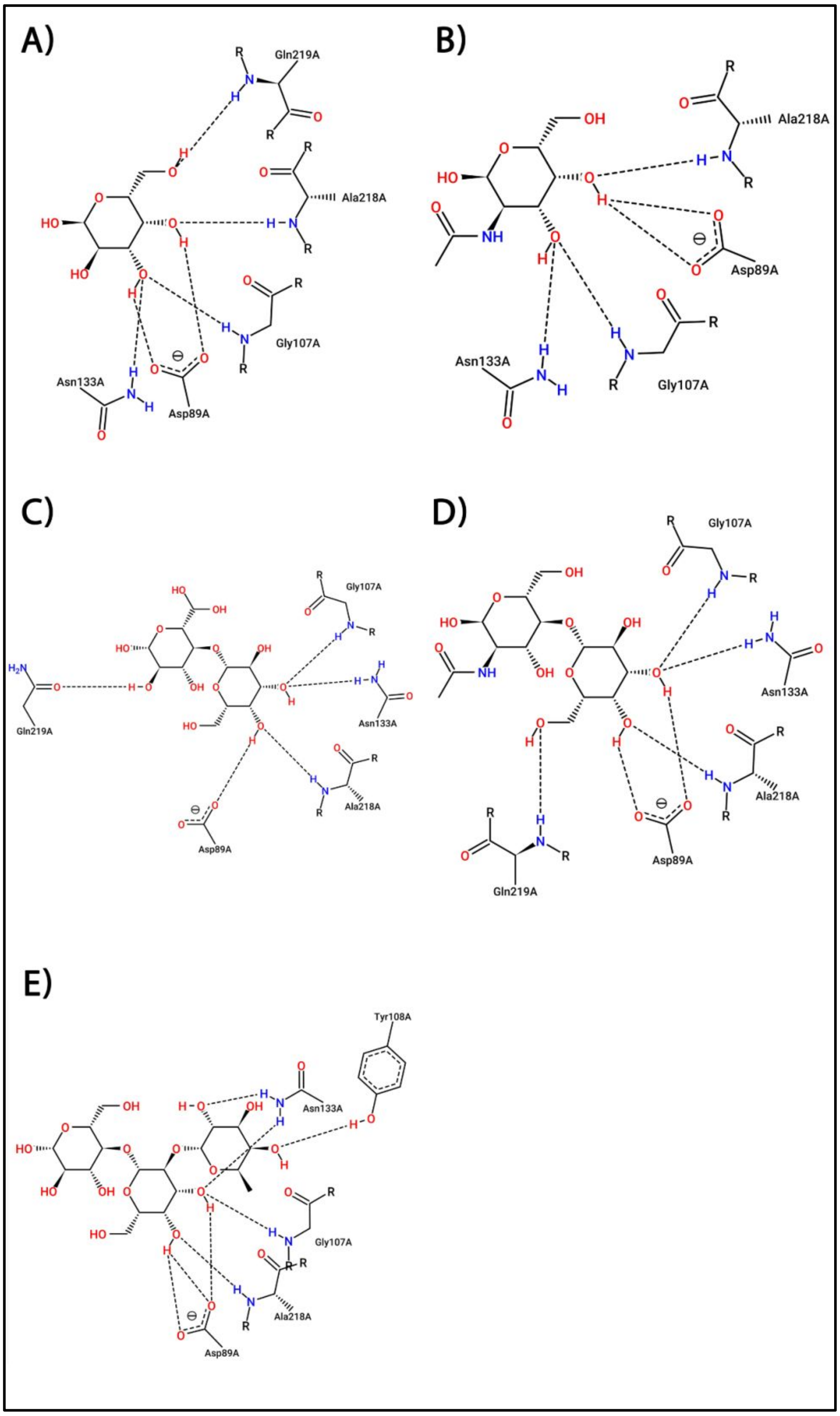

Figure S3. Two-dimensional interaction diagrams of EFusL with tumor-associated O-glycan antigens. (A) EFusL–Tn antigen complex. (B) EFusL–TF antigen complex. Dashed lines indicate hydrogen-bond interactions predicted by molecular docking. The main EFusL residues contributing to ligand recognition are labeled, including Asp89, Gly107, Asn133, Ala218, and Gln219. The diagrams show that both antigens interact with the conserved EFusL carbohydrate-recognition region, mainly through polar contacts involving the terminal GalNAc/Gal residues.

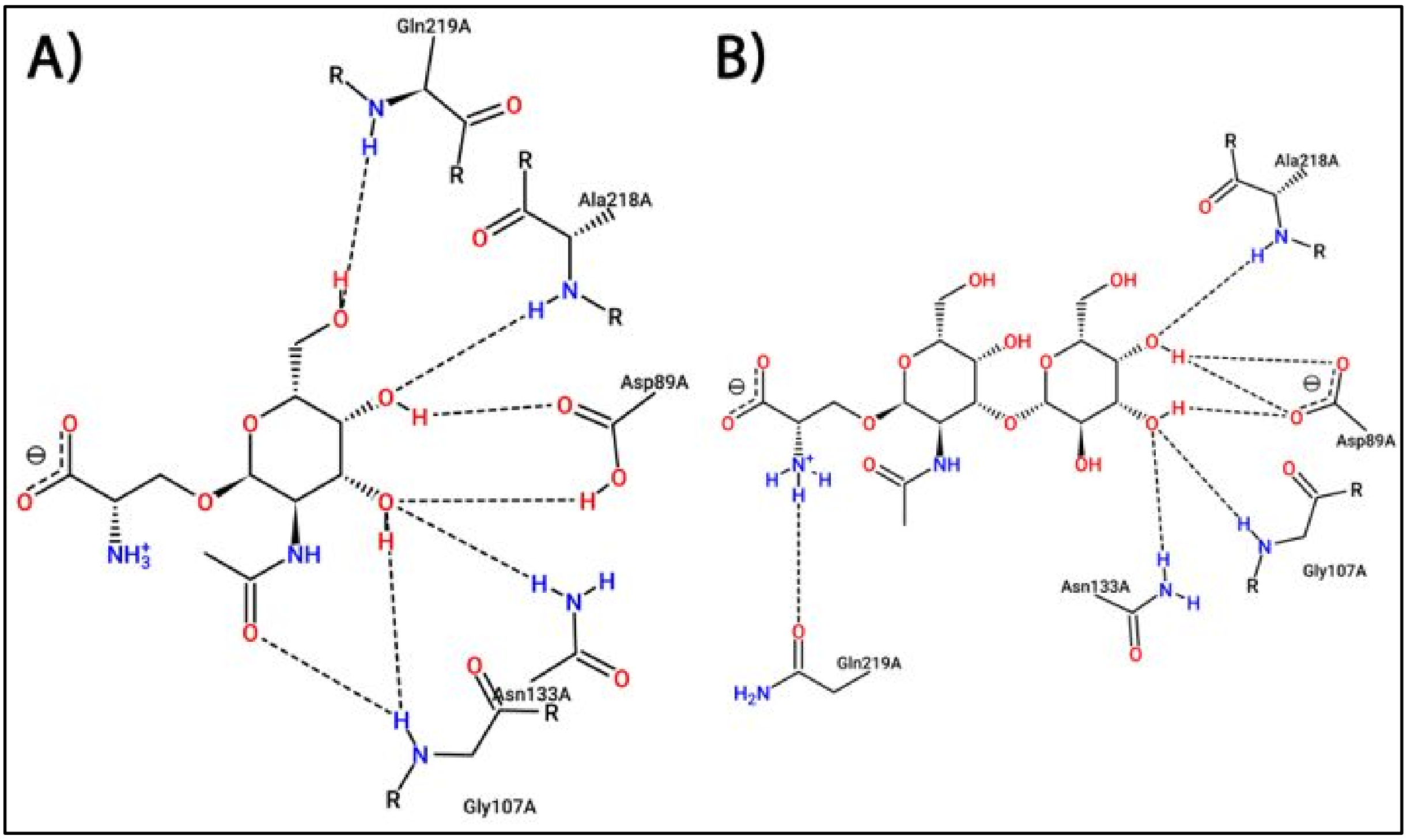

Figure S4. Complete effect of EFusL on the viability of human cell lines. HeLa, HT-1080, and NHDF cells were treated with EFusL at concentrations ranging from 7.8 to 125 µg/mL for 24, 48, and 72 h. Cell viability was assessed using the PrestoBlue™ HS assay and normalized to untreated cells, which were considered 100% viable. Data are expressed as mean ± SD of three independent biological experiments, each performed with four technical replicates. Statistical comparisons were performed against untreated cells within each panel, with Holm-adjusted p-values indicated when significant.

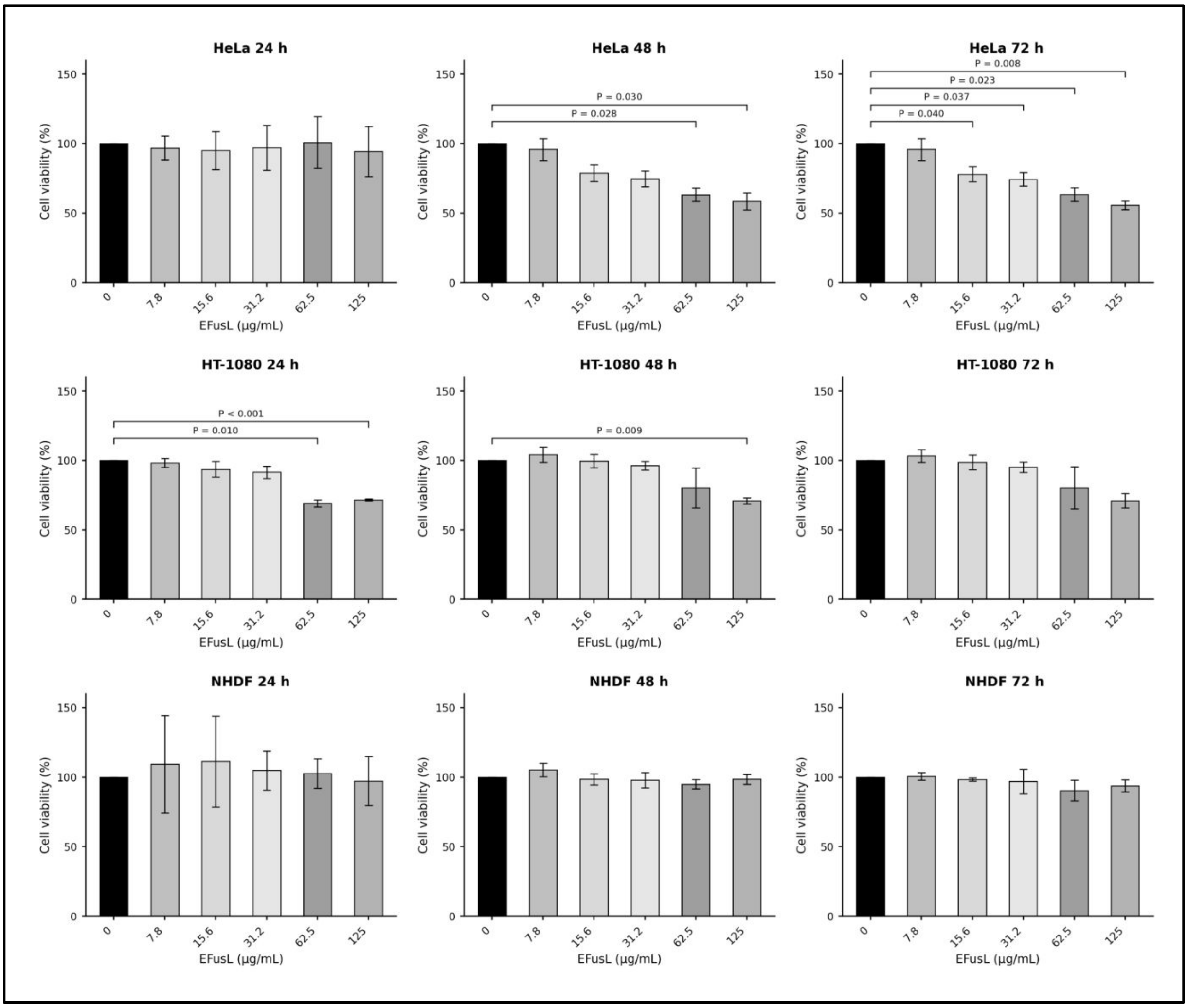

Table S1. Quaternary structure of *Erythrina* lectins. Reported native oligomeric states and subunit molecular masses compiled from original studies; blank entries indicate not reported. EFusL data obtained in this study are included for comparison.

**Lectin**	**Apparent molecular weight**	**Number of subunits**	**Approximate total molecular weight**	**References**
EAL	30 kDa	2	57 kDa	(Ortega et al. 1990)
EArbL	28 kDa	2	58 kDa	(Bharracharyya et al. 1986)
ECA	26 e 28 kDa	2	56,8 kDa	(Iglesias et al. 1982)
EcafL	30 kDa	2	59,3 kDa	(Lis et al. 1985)
EcorL	28 kDa	2	60 kDa	(Lis et al. 1985)
EcostL	27,2 kDa	2	56 kDa	(Perez 1995)
EEL	27 kDa	2	56 kDa	(Gerardo 1984)
EiLL	30 e 33 kDa	2	58 kDa	(Konozy et al. 2002)
EiSL	30 e 34 kDa	2	66,2 kDa	(Horejsí et al. 1980)
ElatL	32 kDa	2	61,9 kDa	(Lis et al. 1985)
ELL	26 e 28 kDa	2	57 kDa	(Bharracharyya et al. 1986)
ElysL	28 kDa	2	58,8 kDa	(Lis et al. 1985)
ERL	29,5 kDa	2	62 kDa	(Pena et al. 1988)
ESL	30 kDa	2	62 kDa	(Kuku et al. 2012)
EspcL	27,6 kDa	2	58 kDa	(Konozy et al. 2002)
ESubL	28 kDa	2	59 kDa	(Bharracharyya et al. 1986)
EvarL	33 kDa	2	51 kDa	(Yamasaki et al. 1992)
EvespL	32 kDa	2	59 kDa	(Kortt 1986)
EVL	31,6 kDa	2	56 kDa	(Moraes et al. 1996)
EPL	25 kDa	2	50 kDa	(Quesada et al. 1998)
ESteL	25 kDa	2	50 kDa	(Quesada et al. 1998)
**EFusL**	**29 e 31 kDa**	**2**	**60 kDa**	**THIS WORK**

Table S2. Primary structure of *Erythrina* lectins. N- and C-terminal sequences (one-letter code) compiled from original reports; blank cells indicate not reported, and "Xaa" denotes an unidentified residue. EFusL terminals determined in this study are included for comparison.

**Lectin**	**N-terminal**	**C-terminal**	**References**
EcafL	VETISFSFSEFEPGN		(Lis et al. 1985)
EcorL	VETISFSFSEFGPGN	AAETHDVYSWSFQASLP ETN	(Adar et al. 1989)
ECA	VETISFSFSEFEPGN	AAETHDVYSWSFQASLP ETN	(Lis et al. 1985)
EflaL	AETISFSFSEFEPG		(Lis et al. 1985)
EHL	VETISFSFSEFEPGN		(Lis et al. 1985)
EiLL	VETISFSFSEFE		(Konozy et al. 2002)
EiSL	VETISFSFSEFE		(Konozy et al. 2002)
ElatL	VETISFSFS		(Lis et al. 1985)
ElysL	VETISFSFSE		(Lis et al. 1985)
EPL	VETISFSFSKFEAG		(Lis et al. 1985)
ESL	VETISFSFS		(Lis et al. 1985)
EvarL	VETISFSFSEFEAGNDXaaLT	AAETHDVYSWSFHASLP ETN	(Yamasaki et al. 1992)
EVL	VETIPFS		(Moraes et al. 1996)
EvespL	VETISFSFSEFEAGNDSLTLQGA ALI		(Kortt 1986)
EZL	VETISFSFSEFEPGN		(Lis et al. 1985)
**EFusL**	**VETISFSFSEFEAGN**	**AAETHDVYSWSFHASLP ETN**	**THIS WORK**
